# Exploration of Emotion Perception in Serious Interactive Digital Narrative

**DOI:** 10.1155/2022/8160695

**Published:** 2022-06-28

**Authors:** Jie Zhang, Yaqian Liu

**Affiliations:** School of Design, Jiangnan University, Wuxi 214122, Jiangsu, China

## Abstract

The procedural process of children's emotional involvement in the interactive digital narrative conforms to children's emotional attachment to the story and enhances the mediating nature of learning through forced interactivity. This study explores how compelling arcs influence learners' preference for serious story content by using a combination of natural language processing methods and statistical analysis methods. By analyzing 474 Chinese short serious stories, the emotional trajectory of each story is generated. Then, the obtained trajectories are combined into clusters of serious story emotional groupings through supervised learning. The study results found that the emotional arc in serious stories can be divided into six basic shapes, and the serious story with the highest preference is the “N”-shaped emotional arc. Emotional ups and downs characterize the emotional narrative aspect of this type of serious story as the story progresses but with an apparent emotional uptick towards the end of the story. Based on experimentally derived emotional topology and narrative generation methods, this paper proposes the design strategies for future emotional arcs to apply to serious interactive digital narratives.

## 1. Introduction

Storytelling is an essential way of communicating thoughts and ideas, going through stages of development from spoken language to written records. At all stages of its development, it is an important art form that inspires human emotions. This makes readers have an attachment to the world depicted in the story. In reading, readers are emotionally attached to the story that matches their current psychology [1]. Similarly, reading is accompanied by the process of continuous satisfaction of psychological needs and gradually develops a knowledgeable and confident self-identity. These psychological needs are mainly expressed as an emotional will, and interaction plays a vital role in satisfying the emotional will. Interactive digital narrative systems provide a more comprehensive storytelling process that integrates systems, users, processes, and frameworks [2]. This technology may generate a customizable narrative environment for users through prototype tales, narrative design, and narrative vectors. Interactive digital storytelling sits at the crossroads of the humanities and computer science study domains, integrating computational storytelling and generative systems, with a great potential for varied applications and aesthetic approaches [3]. It resolves the contradiction between linear narrative and interaction in a “system-interaction-output” way, [4] producing instantiated narrative products through participatory process experiences. Serious Interactive Digital Narrative creates knowledge and wisdom in a specific context by harnessing the strengths of interactive storytelling to construct and convey serious messages. For example, “Global Conflicts: Palestine” is a serious educational game in which the users learn about the natural world by interacting as a journalist and forming a narrative report during the game. This approach provides children with new learning opportunities that are different from the classic linear narrative and is helpful for children's cognition and learning.

Some serious cultural knowledge imparted to readers should not simply be compiled into compact truths but should be conveyed in a serious narrative. The content of serious stories is a module of social reasoning and environmental knowledge, and most of them show the behaviour of human character: aspects of integrity, honesty, and perseverance. The main reason why a serious narrative can move people more than “information” is that the story has the triggering power of emotion, and the microscopic individual narrative carries the life and cultural conditions experienced by the nation's history, highlighting the national solid narrative tendency. Invoking the concept of emotional arcs in narratives, transforming emotional fluctuations into time-series data, is closely related to narrative structure. Whether it is an early automatic storytelling system based on emotional arcs and symbolic planning [5] or a neural story generation method that models emotional trajectories with the support of deep learning techniques [6], they both show that introducing emotion into plot construction results in diverse and exciting stories. By introducing emotion into constructing a serious interactive digital narrative system, personal stories are shaped by the trajectory of emergent emotions. Narratives are organized by emotional experiences, in which personal intentions, goals, and hopes are expressed, and the resulting personal stories contribute to the construction of personal meaning and self-identity. From the perspective of data science, this study takes the texts currently applied to serious narratives as examples to explore which narratives are more acceptable to the readers under the influence of emotional arcs. Furthermore, a future-oriented design strategy for serious interactive digital narratives is proposed based on the experimental results.

Section 2 provides a comprehensive introduction to previous related literature research, Section 3 presents the processing of datasets and sentiment arc analysis methods based on natural language processing and statistical methods, and Section 4 points out the design strategies for emotional arc-perceived serious interactive digital narratives based on experimental results, Section 5 discusses the limitations of the study, and Section 6 summarizes the conclusions and future work.

## 2. Related Work

### 2.1. Serious Interactive Digital Narrative

The narrative itself is a storytelling artifact consisting of a sequence of narrative streams of events that take place in time and space that move the story forward [7]. The interactive digital narrative is an expressive narrative form in digital media; it also is conceived of as a computer system comprising possible storylines [4]. As a systematic framework, the interactive digital narrative has three components, including the drama manager, user model, and agent model [8]. This paper only reviews the research part related to narrative generation in the serious interactive digital narrative.

Interactive digital storytelling has been around for nearly 30 years. In terms of narrative generation, early interactive digital narratives relied on intelligent planning, programming the content of interactive digital narratives as program nodes, and abstracting them into planning problems. Ensure a coherent experience by balancing a coherent story progression and user autonomy in a systematic way [9]. For example, the children's book series “choose your adventure” splits the experience into interaction points with decision-making. Computer intervention in the interactive narrative attempts to use the model of artificially written narrative program node with a model generator based on planning domain language [10]. For example, the Automated Story Director, [11] Player-Specific Stories via Automatically Generated Event (PaSSAGE) [12], Player-specific Automated Storytelling (PAST) [13], these models are based on a specific story (The RED), programing narrative into nodes, study and explore author intent, virtual agent and user modeling in interactive digital narratives to generate emergent narratives, and automatically generate narrative content by modeling drama management, AI experience managers. The researchers further pointer out the possibility of interactive storytelling in the fields of entertainment, training, and education [11]. However, this kind of interactive digital narrative with computer planning as the core is still subject to the strong story script written in advance by the author/designer and the narrative plan that needs to be foreseen in advance, which can only operate in the predefined field, and it is difficult to generate a more complex narrative in line with the human cognition.

This problem has been mitigated by the increasing sophistication of machine learning techniques, and the training of large-scale neural language models has brought more attention to computers' understanding, telling, and creating stories. Computational narrative generates coherent story content through a story-generation approach based on common-sense reasoning; for example, the transformer-based large-scale language model GPT-2 [14] generates fluent text by training on the WebText corpus, a collection of texts scraped from the internet. The computational narrative is not only about the generation of commonsense knowledge but also about the controllability of story generation. Model Fusion improves the quality of randomly produced stories by combining a convolutional sequence to sequence model with a self-attention mechanism.

With the development of computational storytelling, the possibility of interactive digital storytelling applied to serious and non-entertainment environments has emerged. Lugmayr et al. originally defined serious storytelling as storytelling with a purpose beyond enjoyment and identified applications of serious storytelling in fields such as education [7]. The first attempt in this field was serious games, aiming to convey serious messages through constructing the digital media of games. James Lester et al. have been actively studying the construction of a catalog of narrating-centric learning environments in serious games [15]. These include the influence of story on the learning process and offering automated evaluations and individualized feedback from a data-driven perspective. Most of these studies are from a pedagogical perspective and are beyond the current focus of this paper. The SIREN project has not completed the final design and pilot study [16]. However, the study proposes using the computational emotional player model as the pillar of the generation system to ensure an efficient adaptive narrative experience for users, which can provide a reference for our study. Arash et al. focused on player modeling in serious interactive digital narratives, explored how to use artificial intelligence technology to provide personalized experiences to consolidate the influence of serious discourse and proposed a prototype for the game design.

### 2.2. Emotional Arc in Storytelling

Emotion is a vital element of storytelling, a component of the cognitive portion of the tale that inspires in the listener, who sees the narrative as an emotional experience. With the increase of computer power, individuals begin to analyze this emotional experience from the standpoint of big data. Moreover, the current breakthroughs in natural language processing and computational narratology make it feasible to examine the emotion of the text. From the standpoint of computational narrative, Chen et al. have proved that emotional development is important for story understanding [17].

Kurt Vonnegut was one of the first researchers to use data science to analyze the emotional content of stories. He coined the term “emotional arc” in the narrative. He defines the emotional arc as the relationship between the horizontal storytime “start-end” and the vertical emotional “Ill Fortune-Great Fortune.” By identifying emotional arcs in a sample of 1327 stories in the Project Gutenberg fiction collection, Reagan et al. discovered a set of six fundamental emotional arcs that are also the basic building blocks of complicated emotional trajectories [18]. Del et al. derived emotional arcs for each film by analyzing 6,174 films using a combination of natural language processing and econometrics. By plugging these emotional clusters into the econometric model, it is concluded that films affected by the U-shaped emotional arc can achieve box office success regardless of genre, helping to improve the productivity of the entertainment industry [19]. On the opposite, namely, by beginning with the emotional arc and contributing in the production of fascinating stories. During the planning language coding period, Sergio Poo et al. created the interactive narrative experience manager PACE, which generated narrative nodes by rewriting the ballet story Gisele, used PACE to predict the user's emotional response in the narrative process, and used the emotional response to shape the subsequent narrative content, so as to keep the user on the target emotional arc. In the era of computational narrative, EC-CLF, a story generation method based on the emotional arcs of the protagonist proposed by Brahman and Chaturvedi, allows users to present the story's progress by entering the title and the emotional arc of the protagonist [20]. The ViNTER (Visual Narrative Transformer with Emotional arc Representation) model proposed by Kohei et al. can automatically generate image narratives represented by “emotional arcs” as time series. The above researches have analyzed the primary form of an emotional arc and the advantages in the creative entertainment industry from data science. Computational narrative studies the impact of emotional arcs on narrative generation from the perspective of text and image content generation. However, in these studies, the generation of narrative lines still relies on the emotional arc input by the user in advance, and there is a lack of pre-research on the emotional arc of user preferences. The application of this method in the serious interactive digital narrative is difficult to balance the needs of narrative consistency and perceived self-agency. Users are given too much autonomy, and it is difficult to accomplish the educational purpose of narrators in a serious interactive digital narrative. At the same time, the emotional arc does not intervene and influence the narrative content in the narrative process, which is difficult to reflect the positive influence of the process exploration of interactive digital narrative on learners' acquisition.

Through the systematic review of narrative generation in interactive digital narrative and the analysis of serious interactive digital narrative used in serious non-entertainment occasions, the paper combines the computational generation of emotional arc in data science and the application of emotion arc in narrative generation. A two-stage study of our approach to serious interactive digital tales is offered. (i) In the first stage, natural language processing and sentiment analysis are used to acquire the emotional topology of serious narratives based on emotional arcs, and the emotional arcs that learners favor based on the quantity of reading and likes are obtained. (ii) In the second step, we construct our design approach for serious interactive digital tales based on the existing narrative creation methodologies and the emotional topology established in the first stage.

## 3. Method

### 3.1. Dataset

**Figure 1:**
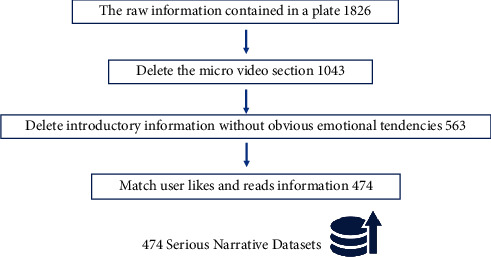
Methods of data processing and cleaning.

The dataset for this project comes from a general application, and there is a separate section in the application called “Red China.” The serious narrations below the catalog are mainly dominated by typical cases, historical figures, and wonderful stories from different periods, comprehensively demonstrating the connotation of the national spirit in Chinese culture for publicity and education. The number of reads and likes for each serious narrative after reading is also available. As a learning platform, Learning Power has collected a lot of knowledge information, including documentaries, concept explanations, micro-videos, and classic works. For this project, we focused on the Serious Stories section, which contains 1,826 pieces of information, in order to filter the quality and dependability of serious tale text material and assure the link between narrative text and learner choice. Deleting the section of the narrative material that only comprises micro-videos decreases the total number of serious tales to 1,043 and then deletes serious stories without clear emotional inclinations, resulting in 563 results. Finally, the dataset is linked with the user reading information on serious tales from the site, including readings and likes. Matching and further cleaning the data resulted in a final dataset of 474 serious stories in total, as shown in Figure 1.

### 3.2. Methodology

**Figure 2:**
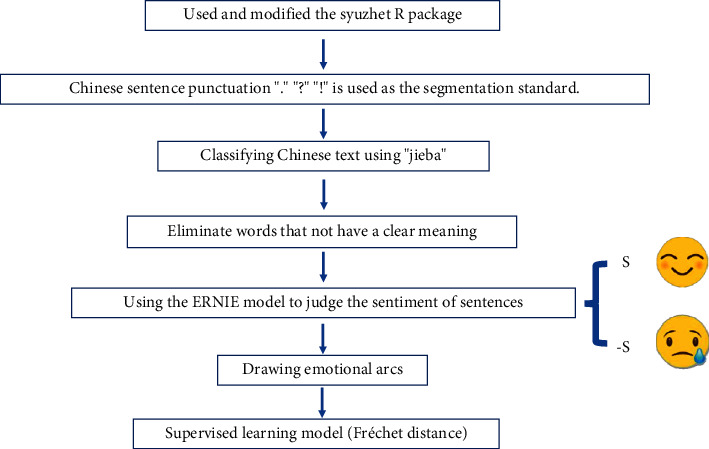
Sentiment analysis and emotional arc drawing methods.

We use the resulting dataset of 474 serious stories for sentiment analysis, and the specific methods used are shown in Figure 2. To analyze Chinese sentiment arcs, we used and modified the syuzhet R package to uncover the underlying structure of narratives through sentiment analysis in Chinese, revealing sentimental changes in narratives that are proxies for narrative movement between conflict and conflict resolution. Unlike the sentiment analysis method based on the sentiment dictionary used by Reagan et al., this study uses the text sentiment analysis method based on deep learning. First, the collected data is preprocessed in a loop, and the Chinese sentence punctuation “.” “?” “!” is used as the segmentation standard. Based on regular expressions, fine Chinese sentences are divided into *n* different sentences for each short story. Then, the jieba word segmentation tool is used to divide each sentence. In word segmentation, the Chinese public stoplist eliminates words that appear frequently but have no clear meaning. The embedding vector of the spliced sentence is obtained through the ERNIE model [21]. The embedding vector is linearly transformed and classified into two categories to judge its emotional tendency. According to the content of the text, the system gives the corresponding scores of positive and negative emotions as *s*; when it is determined to be a positive emotion, it outputs *s*. When it is determined to be a negative emotion, the output is −*s*, and the range of positive and negative emotions is scaled to [−1, 1]; treat each sample in the dataset as a dyad <*d*, *s*>. This study uses a window-based approach to analyze the sentiment of each serious narrative, first extracting the text covered by a separate window, and each time the loop is executed, the window slides forward until the end of the text is reached.

The generated emotional arcs are clustered according to the existing research on emotional arcs in novels and movies by Regan and Marco et al. [18, 19]. We hypothesized that the emotional arcs of serious narratives conform to the six emotion categories studied by Regan et al. The six categories are Rags to riches (rise), Riches to rags (fall), Man in a hole (fall-rise), Icarus (rise-fall), Cinderella (rise-fall-rise), and Oedipus (fall-rise-fall). The resulting 474 emotion arcs were clustered using supervised learning. Specifically, the Fréchet distance algorithm calculates the maximum difference between the two sequences and aligns each generated emotional arc with the six emotional arc templates calibrated in advance. Statistical analysis was then used to introduce the ratio of likes (a number that reflects learners' affirmation of a serious narrative after reading) to the number of readings. The likes rate of serious narrative users under different emotional arc categories is calculated separately. In this way, the learners' preference for serious narratives of different emotional arcs is known.

### 3.3. Result

**Figure 3:**
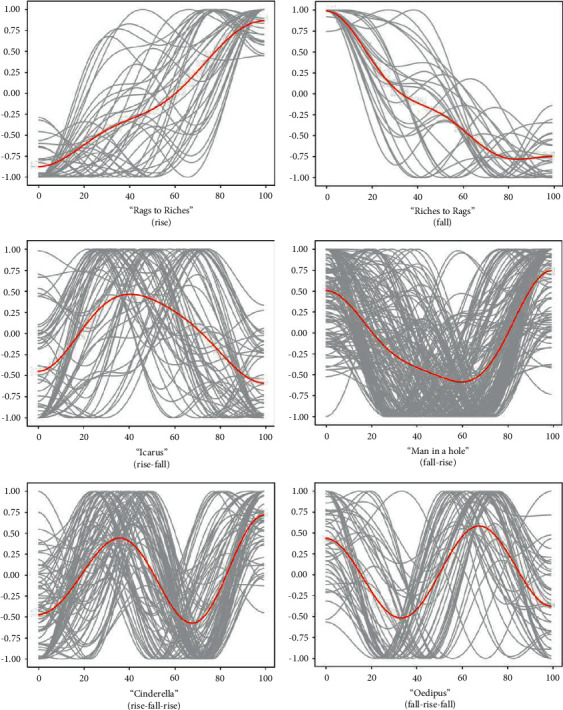
Six emotional trajectories of serious narrative.

**Figure 4:**
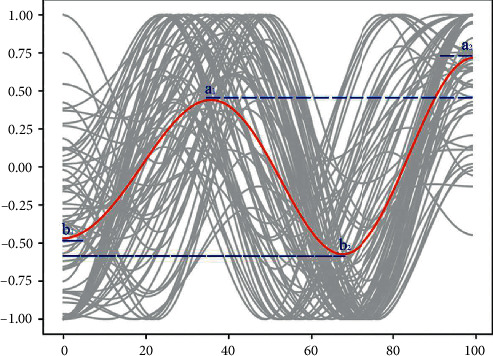
Node analysis of “cinderella” emotional arc.

Through systematic analysis using supervised learning, it is found that all 474 emotional arcs of serious narratives in the dataset can be matched with the six basic emotional arcs proposed by Regan et al. All serious narrative story texts can be divided into six main emotional trajectories.  Figure 3 shows the clustering results of the experiment, where the red line represents the mean of each clustering result.  Figure 4 shows a collection of six arc means. In each figure, the length of serious narration from beginning to end is displayed between the horizontal axis [0, 100]; and the change of emotion is displayed between the vertical axis [−1, 1].

Our 474 serious narrative screening datasets include: “Rags to riches” (rise) 46, “Riches to rags” (fall) 35, “Man in a hole” (fall-rise) 176, “Icarus” (rise-fall) 64, “Cinderella” (rise-fall-rise) 90, and “Oedipus” (fall-rise-fall) 63. There are more than 30 in all categories, and the rest of the summary statistics are shown in Table 1. Detailed serious narrative topics and related information have been stated in Data Availability. According to the data analysis in Table 1, among the six categories, “Man in a hole” and “Cinderella” are the two types of clusters that account for the largest proportion. The reading volume of users is relatively obvious. It is not hard to find common ground between these two emotional arcs in that no matter how the story develops, their results are positive. However, the number of likes after reading can more intuitively reflect the learners' feedback on the story's content after reading from a data perspective. Comparing the average like rates of the six different types of serious narratives found that the Cinderella cluster was the most popular, far exceeding serious narratives influenced by other emotional arcs. As shown in Figure 4, the main feature of this emotional arc is the ups and downs in the middle story but a clear emotional rise at the end of the story. Furthermore, the negative feeling *b*2 of plot emotions in the middle of the story is lower than the negative value at the beginning of the story, and the positive value at the end of the narrative is higher than the positive value in the development process story. By analyzing these six arcs and their readers' preferences, in general, “Man in a hole” and “Cinderella” emotional arcs, which have a good ending *g* after the story's tortuous development, have higher preferences. In contrast, fewer readers prefer “Rags to Riches,” with rising emotions without twists and turns. Compared with serious narratives with rising emotions, “Riches to Rags” serious narratives with declining emotional arcs are more popular. It may be that the serious narrative of the dataset contains many stories about the protagonist going through a stubborn struggle with fate and finally dying generously. Such stories follow the “fall”-shaped emotional arc in the narrative and, at the same time, can influence readers to achieve the purpose of education. Three groups of serious narratives ending in tragedy, the “Riches to Rags” and the “Oedipus” serious narratives both start with a happy state and end with a sad emotional state, while the “Icarus” narrative start with a sad state and ends with a sad state. Comparing these three types, users have a higher preference for the “Riches to Rags” and the “Oedipus” emotional arcs, while users have a lower preference for the “Icarus.” This result suggests that when a serious narrative ends with a tragedy, readers are more receptive to an emotional arc that begins with a positive state. It allows the reader to start the narrative with joy and a sad ending. Meanwhile, the word count difference in serious narrative content under different emotional arcs is also an important factor influencing readers' preferences. Counting the number of words in each category of narrative content and calculating the average, the average number of words in serious narratives is between 1300 and 1550 words. The popularity of serious narratives is higher when the number is around 1300 words. With 1310 words as a boundary, the preference for more than 1310 words is generally lower than that of the serious narratives with less than 1310 words.

## 4. Serious Interactive Narrative Strategies for Emotional Arc Perception

Artificial intelligence and natural language processing offer many opportunities for generating intelligent feedback in interactive digital narratives, making the overall structure of the narrative personal, more relevant to the reader on an emotional level, and inducing a more profound sense of belonging to the narrative. Based on the findings above, we found that learners prefer a serious narrative emotional arc of “rise-fall-rise.” Under the influence of this emotional arc, we propose a serious interactive digital narrative system for emotional arc perception. It is expected to use artificial intelligence technology to assist the design of interactive narratives in consolidating the influence of serious discourse. The serious interactive digital narrative guided by this emotional arc is not just about the story's content being kept in an “N” (Cinderella) arc. However, it emphasizes that the interaction becomes a decision-making process and changes the narrative flow to achieve important situational goals. It is shifting the serious narrative from an output-centric point of view to a procedural narrative point of view, focusing on how the output process is emotionally oriented. It turns from the narrative itself to the process of its construction and finally generates a serious narrative generation mechanism of “data + human + algorithm.” In the proposed serious interactive digital narrative strategy, the “N”-shaped emotional curve in the results is taken as a reference. In the proposed serious interactive digital storytelling strategy, the “N”-shaped emotional arc in the results is used as a reference. The optional emotional dimension question and answer is generated through programmatic content generation and user data; narrative content is jointly generated through emotional interaction between users and the system at nodes and emotional monitoring of narrative generation. Let users invisibly fit the “N”-shaped emotional arc in the emotional experience of the entire serious interactive narrative. We propose only a design prototype, and its realization still requires the joint efforts of the humanities, computing, and design disciplines.

### 4.1. Program Content Generation

**Figure 5:**
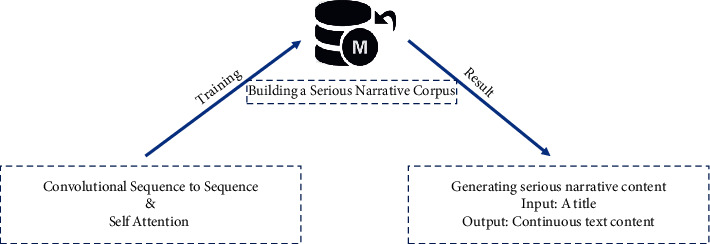
Program content generation.

In order to complete the intelligent content generation of serious interactive narratives, we need to build a large corpus of serious narratives and train the corpus. Ensuring that the generated stories are organized around themes and maintaining the consistency of entities and events in narrative generation using existing text generation methods. The quality of generated stories can be improved by combining the winder sequence to sequence model and self-attention mechanism to ensure that the generated content is the logical continuation of the narrative. Moreover, it eventually reached the state of generating continuous text content related to the input topic, just like the Reddit WritingPrompt [22] project implemented by Fan et al. as shown in Figure 5.

### 4.2. Emotional Dimension Question and Answer

**Figure 6:**
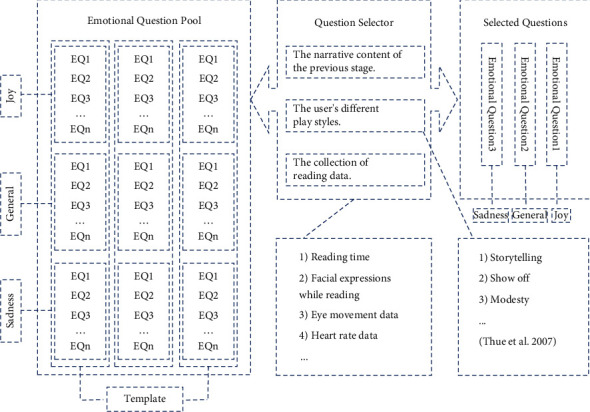
Emotional dimension question and answer.

This part of the questions is organized under specific categories of different emotional levels. The detailed part is shown in Figure 6. Several emotional dimension question templates are preset in the emotional selection pool, corresponding to the three dimensions of joy, general, and sadness, respectively. When narrative generation and interaction occur, the emotion selector modifies the question template in the emotion selection pool at the corresponding node according to the narrative content of the previous stage, the survey of the user's style in the previous stage, and the remote sensing monitoring of the user. The remote sensing monitoring of users during the reading process mainly collects user reading data, including reading time, facial expressions [23] while reading, eye movement data, and heart rate data. The user's style is divided into storytelling, show-off, and modesty. Then, push the question to the user in an orderly manner. In this way, effective emotional questions are selected. An appropriate emotional experience is maintained for users, keeping the serious narrative interesting for the user based on the corresponding emotional arc. The key motivation for this design decision is to focus on delivering several aspects of emotional options for the user's reading experience. The scope and style of questions can be defined according to narrative material and the reader's style. Provide the user with emotional choice while keeping her inside the confines of the narrative structure to produce the effect of the serious story of emotion perception.

### 4.3. Emotion-Driven Program Content Generation

**Figure 7:**
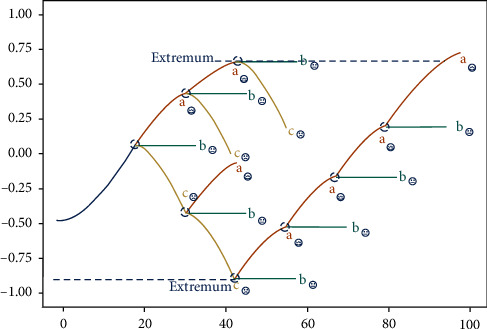
Emotion-driven program content generation.

In emotion-driven program content generation, we need to introduce the emotion monitoring and the emotional reward models, use emotion monitoring to supervise the content generated in the process of program generation, and use emotional dimension questions and answers to complete emotional rewards. The two work together to approximate an “N”-shaped emotional arc. The detailed system process is shown in Figure 7. After the user enters the corresponding topic, the system provides narrative content with rising emotions by default. When the content is generated to a turning point, the emotion selector executes the command to provide the user with emotion questions of three different dimensions. The different choices of users correspond to three possible narrative emotional trajectories: in the picture, *a*-continue to rise, *b*-stay level, and *c*-begin to fall. When the user's choice is “*a*,” the narrative emotion continues to rise until the system determines that the emotional arc reaches the extreme value, and the negative intervention of the narrative content needs to be enforced. When the user chooses “*b*,” it may indicate that the user's emotional state is not high at this time, and a relatively gentle narrative needs to be generated to help the user complete the transition. When the user selects “*c*,” it has begun to enter the negative stage of the narrative. The emotion needs to be questioned again at the node where the narrative progresses until the emotion monitoring detects that the emotional state generated during the narrative process is lower than the negative state at the beginning. At the node of narrative generation, the generation of narrative content is affected by the way “*a*, *b*, and *c*” appear alternately until the narrative generation begins to enter the final emotional rising stage. The emotional Q&A at the node in the final stage should be dominated by positive questions, constantly guiding users to reach the final positive emotional state of the serious narrative, and surpass the positive emotional peak in the narrative process. In a sense, this method achieves the purpose of personalizing the narrative with the user's more preferred emotional arc and reflects the exploration and influence of the user's emotional perception in the serious interactive narrative.

## 5. Discussion

### 5.1. Analysis of the Main Reasons for the Preference for “N” (Cinderella)-Shaped Emotional Arcs in Serious Interactive Digital Narratives

Through the application of natural language processing technology, it is concluded that users have the highest preference for “N”-shaped emotional arcs in serious narratives, and it is concluded that 1310 words are the best word count for serious narrative texts. Our findings are inconsistent with the novel emotional arcs of Regan et al. that “Icarus,” “Oedipus,” and “Man in a hole” are the most downloaded e-books and are more likely to lead to success. There is also a difference with the “U”-shaped emotional arc being more productive in the film industry. This phenomenon may suggest that the non-entertainment purpose of the serious narrative makes the desire for emotional expression different from the entertainment in novels and movies. Reading novels, serious narratives, and watching movies, users experience varying lengths of time. Reading a novel, a serious narrative, and watching a film also take different lengths of time, with the average reader spending hours or even days reading a novel and watching a film. In our dataset, the average word count of serious narratives is 1300–1500 words, and readers only need 10–15 minutes to read these texts. Therefore, the serious narrative guided by “cinderella” is more popular than the movies and novels guided by the emotional arc of “cinderella” may be different from the fact that the background and the foreshadowing can be set up through large paragraphs in novels and movies. A serious narrative needs to express one's mind directly, convey ideas, and achieve the purpose of conveying knowledge in a short period [24]. Under the influence of this reason, people are more willing to experience more emotional narratives with limited text and time. The protagonist's bumpy experiences and ups and downs in the narrative can arouse the readers' emotional resonance. In particular, the endings of the narrative under the emotional arc are more positive, and people are pleased to learn and appreciate the spirit and energy conveyed by the protagonist in such a short period.

### 5.2. The Influence of Selected Text Narrative Research on Multimedia Narrative

Due to the higher availability of the textual content of serious narratives in this study, serious narratives in micro-video, audio, and other media are not included. In the narrative environment of digital and multimedia fusion, the text is still the primary way of carrying narrative content in video, animation, games, virtual reality, and other forms of expression. Video and animation production requires scripts provided by text content, and the completion of games and virtual reality requires the world view and interaction logic of text content architecture. Text is a succession of several phases in the development of narrative material. The research on the emotional arc of textual story will serve as the basis for multimedia research, and utilize text to lead the production of future multimedia tales. The serious story is always about the transfer of information and wisdom, and the study of the emotional arc of serious text narrative as an example will become the emotional support of serious narrative, enabling it to enrich the media world with a completely different narrative approach [7].

### 5.3. Is it Valuable and Meaningful for the Emotional Arc to Intervene in Serious Interactive Narrative

The fundamental contribution of this research is to expand on earlier work in a way that enhances emotional perception, presenting a mechanism that allows individual users to develop growing serious stories through more favored emotional exchanges. This tailored method to story development offers them with an adaptable series of emotional experiences and learning events [25], allowing learners to become knowledge creators in the learning process rather than passive recipients of the information. Such a method can help groups with higher requirements for interactivity in the narrative and are more eager to acquire specific knowledge [26]. For example, it can help children complete the learning of knowledge and the construction of self-cognition in the serious narrative of emotional perception.

## 6. Conclusion

Humans are part of the system in a serious interactive digital storytelling system, and knowledge creation and meaningful interaction are the main goals of human involvement in this system. This research focuses on the emotional arc in the narrative, a fundamental aspect of the story that evokes the audience's cognition. Firstly, the preference degree of learners' emotional arcs in a serious narrative is discussed. In general, learners prefer serious narratives with cheerful endings with twists and turns, and they have the highest preference for “N”-shaped emotional arcs. Nevertheless, when a serious narrative ends tragically, learners prefer a positive beginning regardless of the twists and turns. Our goal is to intervene in serious narratives with readers' preferred emotional arcs and achieve serious contexts. Based on this research combining data science and humanistic perspectives, we propose a serious interactive digital narrative design strategy for emotion perception assisted by the artificial intelligence technology. The elements and methods of emotional arc intervention in a serious interactive narrative are discussed. It is expected to have particular guiding significance for creating a serious interactive digital narrative in the future. The main innovation of this research is to obtain readers' preference for narrative emotional arcs in serious narratives through experiments and then use the experimental conclusions to guide the generation method of serious interactive narratives. It applies emotional arc in a narrative “forward and rear.” This paper proposes a feasible future way of serious interactive narrative driven by emotional arcs that combine existing technology and previous research in the strategy part. For future work, it is necessary to design a serious interactive digital narrative system through practice, and conduct more in-depth research on the effectiveness of serious interactive digital narrative with emotional perception by combining the pilot research methods of qualitative and quantitative measurement and more experimental evaluation.

## Figures and Tables

**Table 1:** Summary statistics of analysis results.

	Rags to riches	Riches to rags	Man in a hole	Icarus	Cinderella	Oedipus
Number	46	35	176	64	90	63
Average reads	24401.7	22246.89	46692.81	36798.40	48236.34	41877.84
The average ratio of reads to likes	3.88%	4.28%	4.42%	4.07%	5.13%	4.13%
Average word count	1397.9	1301.8	1298.7	1547.7	1310.6	1455.3

## Data Availability

The datasets presented in this study can be found in online repositories. The names of the repository/repositories and accession number (s) can be found below: https://github.com/undo123/Serious-Narrative-Dataset.

## References

[1] Alexander K. J., Miller P. J., Hengst J. A. (2001). Young children’s emotional attachments to stories. *Social Development*.

[2] Gil M., Sylla C. (2022). A close look into the storytelling process: the procedural nature of interactive digital narratives as learning opportunity. *Entertainment Computing*.

[3] Roth C., Koenitz H. Evaluating the user experience of interactive digital narrative.

[4] Koenitz H. (2015). Towards a Specific Theory of Interactive Digital Narrative. *Interactive digital narrative*.

[5] Theune M., Rensen S., op den Akker R., Heylen D., Nijholt A. (2004). Emotional Characters for Automatic Plot creation. *International Conference on Technologies for Interactive Digital Storytelling and Entertainment*.

[6] Yao L., Peng N., Weischedel R., Knight K., Zhao D., Yan R. (2019). Plan-and-Write: towards better automatic storytelling. *Proceedings of the AAAI Conference on Artificial Intelligence*.

[7] Lugmayr A., Sutinen E., Suhonen J., Sedano C. I., Hlavacs H., Montero C. S. (2017). Serious storytelling - a first definition and review. *Multimedia Tools and Applications*.

[8] Riedl M. O., Bulitko V. (2013). Interactive narrative: an intelligent systems approach. *AI Magazine*.

[9] Riedl M., Saretto C. J., Young R. M. Managing Interaction between Users and Agents in a Multi-Agent Storytelling environment.

[10] Riedl M. O., Young R. M. (2010). Narrative planning: balancing plot and character. *Journal of Artificial Intelligence Research*.

[11] Riedl M. O., Stern A., Dini D., Alderman J. M. (2008). Dynamic experience management in virtual worlds for entertainment, education, and training. *International Transactions on Systems Science and Applications, Special Issue on Agent Based Systems for Human Learning*.

[12] Thue D., Bulitko V., Spetch M., Wasylishen E. (2007). Interactive storytelling: a player modelling approach[C]//Proceedings of the aaai conference on artificial intelligence and interactive. *Digital Entertainment*.

[13] Ramirez A. J., Bulitko V. Telling Interactive Player-specific Stories and Planning for it: ASD+ PaSSAGE= PAST.

[14] Radford A., Wu J., Child R., Luan D., Amodei D., Sutskever I. (2019). Language models are unsupervised multitask learners. *OpenAI blog*.

[15] Sabourin J. (2011). Affective support in narrative-centered learning environments. *Affective Computing and Intelligent Interaction*.

[16] Grappiolo C., Cheong Y.-G., Togelius J., Khaled R., Yannakakis G. N. Towards Player Adaptivity in a Serious Game for Conflict resolution.

[17] Chen J., Chen J., Yu Z. (2019). Incorporating structured commonsense knowledge in story completion. *Proceedings of the AAAI Conference on Artificial Intelligence*.

[18] Reagan A. J., Mitchell L., Kiley D., Danforth C. M., Dodds P. S. (2016). The emotional arcs of stories are dominated by six basic shapes. *EPJ Data Science*.

[19] Del Vecchio M., Kharlamov A., Parry G., Pogrebna G. (2021). Improving productivity in Hollywood with data science: using emotional arcs of movies to drive product and service innovation in entertainment industries. *Journal of the Operational Research Society*.

[20] Brahman F., Chaturvedi S. (2020). Modeling Protagonist Emotions for Emotion-Aware storytelling. https://arxiv.org/abs/2010.06822.

[21] Sun Y., Wang S., Li Y. (2020). Ernie 2.0: a continual pre-training framework for language understanding. *Proceedings of the AAAI Conference on Artificial Intelligence*.

[22] Fan A., Lewis M., Dauphin Y. (2018). Hierarchical Neural story generation. https://arxiv.org/abs/1805.04833.

[23] Rao N., Chu S. L., Faris R. W., Ospina D. (2019). The Effects of Interactive Emotional Priming on Storytelling: An Exploratory study. *International Conference on Interactive Digital Storytelling*.

[24] Raybourn E. M. (2014). A new paradigm for serious games: transmedia learning for more effective training and education. *Journal of computational science*.

[25] Moradi-Karkaj A. Serious interactive digital narrative: explorations in personalization and player experience enrichment.

[26] Fan M., Fan J., Jin S., Antle A. N., Pasquier P. EmoStory: A Game-Based System Supporting Children’s Emotional Development.

